# Clinical Observations in Surgery Made in the Hospital of Instruction at Strasbourg

**Published:** 1840-07

**Authors:** 


### ART. III.

*Clinique Chirurgicale de l'Hôpital d'Instruction de Strasbourg.* Par P. MALLE, Professeur d'Anatomie, &c.—*Paris,* 1838. 8vo, pp. 756.
*Clinical Observations in Surgery made in the Hospital of Instruction at Strasbourg.* By P. MALLE, Professor of Anatomy and Physiology, &c.

THE practical works of military surgeons rarely admit of a complete or regular analysis. They usually contain only the records of the cases which were subjects of particular interest to those under whose observation they occurred, and the task of the reviewer is therefore limited to the selection of the " optima ex optimis," and the consideration of such general modes of practice as are not commonly inculcated. With these views we have examined the work of M. Malle, one of the best of its kind, and in many respects so good as to induce regret that it has not been published in a form that might admit of more minute analysis.

1. The first case of which we shall make an abstract is a remarkable one.

*Wound of the Heart.* A soldier, æt. thirty-one, was amusing himself with a comrade in firing, when suddenly his friend's gun burst, and wounded him. He fell instantly in syncope, but soon regained his senses and complained of a severe pain behind the sternum. He was carried to the hospital, where he presented the following symptoms: About two inches on the inner side of the left nipple, between the sixth and seventh ribs, there was a small wound, which gave passage neither to blood nor to air. The chest was normally resonant beneath it; the patient had some bloody expectoration; the heart's motions were obscure and the pulse feeble; there was dyspnœa; the skin was cold, the face pale, and the patient felt as if he should faint. The surgeon in attendance regarding the greater part of these symptoms as the effect of the fright, ordered some simple means. Four hours after reaction took place, and the patient was bled to ten ounces, after which the expectoration of blood completely ceased. The pulsations of the heart, however, remained obscure, the pulse was small, and the sternal pain continued; but the face had regained its colour, the heat of the surface had returned, and the threatenings of fainting had passed away; he was bled again in the evening.

Next day, the patient had passed a tranquil night; the pulse was fuller and regular, at 100; the face flushed but expressive of suffering; the pain behind the sternum continued; the ear distinguished at the part a kind of undulatory crepitation, something like that heard in a varicose aneurism; and there was a little crepitation in the left lung near the heart. Everywhere else the natural respiratory murmur was heard, though the dyspnœa continued; the horizontal position was irksome. The patient was again bled to ten ounces, and cupped over the heart. On each of the two following days the condition of the patient remained unaltered, and the same depletory means were repeated.

On the fourth day after the accident he was better; he had slept four hours and had lost his faintness. He had less pain, there was no longer any râle, the pulse was less frequent and regular. The improvement continued during the succeeding days; the patient had some appetite and took some light food. The pain behind the sternum was almost gone; but there was still dulness in the precordial region: the pulse remained feeble, and he did not evidently gain strength. His apparent convalescence, though it advanced slowly, would probably have been more marked had it not been twice or three times interrupted by the patient's heat of temper and refusal of being restrained to perfect quietude. After each fit of anger his cough became worse, the dulness in the precordial region more extensive, the pain at the sternum more acute, and the pulsations of the heart more obscure; and for every such aggravation of symptoms depletions were required and were generally efficient. He continued thus alternately improving and suffering from a return or aggravation of his first symptoms up to the forty-second day after the accident, when his condition was such as to permit a hope that his recovery would be permanent. At this time, however, he was seized without any evident cause with erysipelas of the left leg, with fever, &c.; his old symptoms returned with irrepressible violence, and he died on the 14th of May, having received the wound on the 28th of March.

CHARITY HOSPITAL; MEDICAL LIBRARY.

On examination, the brain and the abdominal organs were found healthy. In the chest, at the wounded part, there was a cicatrix scarcely firm. The right lung presented on its anterior surface and near the heart a small cicatrix which was also discoverable under the corresponding portion posteriorly, proving that the lung had been perforated. It was also hepatized for about four inches, and united by some slight adhesions to the pleura. The pericardium was larger than usual, and at first appeared distended with liquid. It contained about five ounces of reddish sanies, and some fibrinous clots, two of which adhered to the heart. A foreign body was fixed in the left ventricle. On closer examination this was found to be a portion of the stock of the gun that had burst; it was situated at the front and about the middle third of the ventricle; its free extremity, which was about as large as a full-sized writing quill, projecting about ten lines; the cavity of the ventricle contained a very firm coagulum extending into the aorta. The piece of wood had traversed the left ventricle and the septum, and projected into the cavity of the right ventricle. Its direction was obliquely from without inwards and from below upwards; its form was somewhat triangular, tapering irregularly from the part that projected in front to that which traversed the septum. The internal surface of the heart was red, and in parts a little softened, especially near the apex; near the valves, on the contrary, the membrane appeared slightly hypertrophied. (p. 274.)

Although instances of recovery from slight wounds of the heart are now far from rare in the records of surgery, yet we believe this is the most severe injury of that organ which has not proved fatal in a few days or hours. In the case by Mr. Davis in the "Trans. of the Prov. Med. and Surg. Association," vol. ii., which in the mode of its production was nearly similar to this, the patient lived only five weeks, and in that case the right ventricle alone was injured. In all the other cases of severe injury death ensued at much earlier periods. The character of the injury in this instance confirms the opinion generally received that the rapidity of death is directly proportioned to the quantity of blood effused, but particularly to the quantity which is retained in the pericardium and presses upon the heart; so that when, as in this case, the aperture is blocked up, either by the foreign body or by a coagulum, life may be considerably prolonged. In its symptoms, also, this example confirms the result of former experience that there are no important injuries less clearly indicated by their immediate effects, or of which all the signs are more inconstant than wounds of the heart. We observe, in a late number of the Archives Générales de Médecine, that M. Jobert believes, from some cases that have occurred to him, that the only symptoms of penetrating wounds of the heart that can be depended upon are a sound similar to that heard in a varicose aneurism, which continues till the wound is plugged up by a coagulum, and a constant convulsive commotion of the heart's fibres, which continues even after the formation of the coagulum. In the present case, the first and the clearest of these symptoms was observed, although the wound was nearly closed.

2. M. Malle's next most important case is a *Dislocation of the Carpus forwards.*

A soldier, while tipsy, threw himself from a second floor into the street. He fell, to all appearance, partly on the palm of his right hand, and partly on his head. When carried to the hospital he had all the symptoms of severe concussion. The hand appeared at first sight considerably deformed; it was extended on the forearm, and the carpo-metacarpal region appeared shortened; at the anterior part of the joint there was a remarkable projection, a little convex transversely, and apparently formed by the carpus. The radius and ulna were placed behind; the former preserved its length, and as usual extended beyond the ulna. The fingers were flexed, the tendons of the flexor muscles appearing very tense. With considerable difficulty the dislocation was reduced, but the patient died of the effects of the concussion three days after the fall.

All the parts around the wrist were found engorged and infiltrated with blood. The anterior ligament of the capsule was ruptured; the lower extremity of the radius was unbroken, but the bones of the first row of the carpus were unnaturally moveable upon one another; the ligaments uniting them seemed to have suffered from some violence, but there was no trace of fracture. At the posterior or dorsal part of the wrist there was a similar bloody infiltration of the soft parts, but no injury of the bones. (p. 605.)

The rarity of this accident is proved by Dupuytren and Sir A. Cooper not having met with it. Boyer and Dessault each saw one case of the same kind; and there is an example of it in the museum of St. Bartholomew's Hospital.

3. *Softening of the posterior part of the spinal cord, with loss of motion in the upper extremities, and persistence of their sensibility.* A corporal, æt. twenty-four, came to the hospital in March, 1828, with loss of motion in the upper extremities. He had fallen in his youth from a considerable height on his back, and had felt at that time a severe pain in the dorsal region of the spine. A few days' rest, however, removed this, and he returned to his play. From that time, however, he had felt pains at uncertain intervals in the part which was injured, and their severity was increased by moving the spine; but rest still removed them in a few days. At fifteen the pains were more rare; and up to eighteen he could not remember to have felt them. At that time he addicted himself to excessive masturbation; and he remarked that whenever he emitted semen he felt in the cervico-dorsal region pains like that of a pin scratching the deep parts about there. On giving up this habit, the pains disappeared again; and he soon afterwards was called into the army, where he served actively, and was about to be made sergeant, when, after a severe fatigue, he felt such an acute pain in the usual spot that he was obliged to come to the hospital, where cupping, leeching, and blisters almost completely cured him. A vague pain, however, appearing from time to time, still remained; his health was uncertain, and he could not take exercise without suffering often severely; the motions of his arms were soon found difficult, and the least exertion fatigued him. The malady increasing, he again came to the hospital. His upper extremities were emaciated, and the voluntary movements of them almost entirely destroyed, though he could still contract some of the muscles, as the flexors of the forearm; their sensibility was natural. He had a deep but not very acute pain at the lower cervical and upper dorsal vertebræ. Nothing unusual could be discerned externally; pressure, even when forcible, gave but little pain; all the other functions were healthily executed, and there was no paralysis of any other part than of the upper extremities. Cupping and other means were employed, but the patient grew worse, became more and more emaciated, lost all power of motion in his arms, and died in May.

The brain was healthy. The dura mater of the spinal cord appeared elevated at the part where the pain had been felt; its tissue also seemed externally softer and thicker. The medulla spinalis was altered from the fifth cervical to the third dorsal vertebra inclusively; its substance at this part was so soft, that it was like *bouillie;* this softening was greatest at the first dorsal vertebra; it became less and less as the cord was examined more deeply, and ceased nearly at the centre of the medulla. However, it was by insensible degrees that the organ acquired its normal condition; above and below the passage from the morbid to the healthy state was more sudden. The altered portion of the cord, when attentively examined, showed that there were a great number of blood-vessels in its interior; the softening, in fact, was reddish, and as if the nervous substance had been mixed with a certain quantity of blood.

The other organs were generally healthy; the sixth and seventh left ribs were affected with tuberculous softening near the vertebræ. (p. 741.)

This is another of those exceptionable cases to which rather too much importance has been attached of late. The works of Ollivier and others now leave no doubt that there are cases by which, assuming softening of the spinal cord to be destructive of its powers, the opinions commonly received of the distinct functions of its columns and even of its being a conductor of the nervous influence from below upwards to the brain, are distinctly contradicted. Thus, in addition to this and similar cases of paralysis of one function with apparent disorganization of the column appropriated to the other, there are recorded examples of paralysis of one or both upper limbs, with a morbid alteration only in the lower portion of the cord; of paralysis of a limb on one side, with organic change of the opposite columns; of paralysis of only one or two limbs, with softening equally affecting the whole cord; of paralysis of distinct parts of the body supplied with nerves from a portion of the cord seemingly healthy, while other parts supplied with nerves coming from a diseased portion exhibited no impairment of power; and so on. All these cases, of which the number now recorded may amount to some thirty or more, may well bear a heavy weight of doubt on the minds of those to whom rarities, though exceptions, are more attractive than common generalities. But, if fairly considered, these cases destroy each other's importance; if there be anything certain in physiology, it is that when a part of the spinal cord is destroyed, the parts of the body supplied with nerves attached to the lower portion lose their sensibility, and can be no longer voluntarily moved; yet cases are recorded where, with excessive softening of a portion of the spinal cord, the parts supplied with nerves from below were not paralyzed. But these cases, even if they were far more common, could not overthrow the well-established general fact; they only prove, if anything, that softened nervous matter may retain its power of conducting impressions to and from the brain; and they may, therefore, be employed as evidence *against* rather than confirmatory of the apparent contradictions implied in other cases of local softening of the cord, without corresponding paralysis of the parts supplied from the altered portion. The fact is, we do not yet know what conditions of nervous matter render it incapable of performing its natural functions, and therefore these confessedly exceptional cases cannot yet be of any weight against opinions founded on direct experiment or more certain observations. It will be only in accordance with what is commonly observed in the progress of science, if those which now appear exceptions should soon become the strongest proofs of the laws against which they are now adduced.

We pass to the consideration of a few of the more novel practical observations that are scattered through the work.

4. M. Malle doubts the correctness of the assertion commonly made, that the aperture at which a gun-shot enters the body is always smaller than that at which it makes its exit. He was led to this doubt by some cases that occurred to M. Begin, and he endeavoured to solve it by experiments similar to those which M. B. had himself once performed at Val-de-Grâce. Several shots were fired at a number of dead bodies, and in every case it was evident that, at whatever part the ball penetrated, the hole at which it entered was larger than that by which it passed out. The latter was only less regular than the former, and its edges were prominent, and thus distinguished from the rounded and depressed contour of the entrance-hole. Some subsequent cases of men shot during life confirmed these facts, and lead the author to believe that all before his time have been mistaken in their observations. (p. 52.)

5. There are some very excellent observations on the influence which fasciæ and other tough tissues have in directing the course of purulent collections, from which the author takes occasion to point out that the conclusion commonly adopted, of a natural tendency in abscesses to approach the surface of the body, is far too general; an opinion in which, for many facts and reasons, we entirely accord with him. Among the cases to which he alludes in illustration of his remarks, are two in which lumbar abscesses opened in the perineum. (p. 107.) In one case, the matter, descending from the first three lumbar vertebræ, had passed through an aperture in the levator ani, after having made its way through the fascia iliaca and the fascia of the pelvis.

6. M. Malle also (p. 305) combats the opinion which is generally received (and more especially by English surgeons), that in wounds of arteries it is always necessary or very advisable to tie the vessel both above and below the wound. He quotes some good facts in favour of his opinion; and against the usually adopted plan urges its frequent difficulty, as in the case where J. Bell was obliged to remove two inches of the fibula to get at the posterior tibial artery, and in that where Deschamps had to make a cut seven inches long through the muscles of the calf to secure the peroneal artery, and was at last obliged to cut the outer edge of the wound, and pass, at haphazard, two ligatures around a mass of tissue above and below the spot from which the blood issued. The cases in which the ligature of the artery at some distance above the wound was successful were a traumatic aneurism of the ulnar artery and another of the carotid. It is evident that no general rule on this subject can be established; the comparative facilities of the two operations, the extent of anastomosis between the wounded and other arteries, the probable size and character of the wound, and a number of other circumstances, varying in each case, must decide the most appropriate mode of proceeding for each.

7. M. Malle gives in this volume a short recapitulation of the principal facts of his work on the diseases of the sympathetic glands, for the study of which a residence at Strasburg is, he tells us, very favorable. (p. 356.) We are surprised, however, at the result to which his experience has led him in their treatment, for he tells us that the enlargements of the glands seldom disappear after they have attained a certain size; all means are ineffectual, and unless they are quickly removed, suppuration takes place, and the disease is indefinitely prolonged. Extirpation is, therefore, the best remedy, and nothing is more simple than its performance. An incision proportioned to the size of the tumour is to be made over its whole length, and the mass is to be enucleated "rather by tearing than by dissection." Such an operation, even without the occasional inconveniences and dangers which M. M. admits, is a most severe as well as simple one, and happily (though enlarged sympathetic glands are common enough) very rarely resorted to or necessary here. We recommend M. Malle, therefore, in return for the many excellent remarks which he offers in this work to the surgeons of this and all other countries, to take from our works some advice on the treatment of this his favorite disease. We think that in these cases his ardent attachment to the Broussaian physiology of disease is not less inimical to his patients than to a judicious and timely use of tonics.

